# Efficacy and safety of Shenmai injection in the treatment of viral myocarditis: a systematic review and meta-analysis

**DOI:** 10.3389/fphar.2024.1453946

**Published:** 2024-10-25

**Authors:** Qianhe Yang, Jie Qin, Ziyue Li, Yue He, Yabin Zhou

**Affiliations:** ^1^ Graduate School, Heilongjiang University of Chinese Medicine, Harbin, Heilongjiang, China; ^2^ Endocrinology Department, The Fourth Affiliated Hospital of Harbin Medical University, Harbin, Heilongjiang, China; ^3^ Graduate School, Fujian University of Chinese Medicine, Fuzhou, Fujian, China; ^4^ Department of Cardiovascular Medicine, First Affiliated Hospital of Heilongjiang University of Chinese Medicine, Harbin, Heilongjiang, China

**Keywords:** Shenmai injection, viral myocarditis, safety, meta-analysis, systematic review

## Abstract

**Purpose:**

To evaluate the efficacy and safety of Shenmai injection for the treatment of viral myocarditis (VMS) through systematic evaluation and meta-analysis.

**Methods:**

Seven databases were searched to identify randomized controlled trials that examined the use of Shenmai injection for the treatment of VMS. The databases were searched from inception to 20 January 2024. The quality of the included studies was evaluated via the Cochrane risk of bias tool (RoB 2) version 2. Data analysis was performed using Review Manager 5.4 and Stata 16.

**Results:**

In total, 18 randomized controlled trials were included. The trials were conducted in 2006–2024 and included 1,661 patients with VMS. The results reveal that Shenmai injection combined with conventional treatment was superior to conventional treatment alone in terms of the following outcomes: total effective rate [RR = 1.22, 95% CI (1.16, 1.28)], CKMB [SMD = −3.33, 95% CI (−4.85, −1.81)], electrocardiogram (ECG) efficacy [RR = 1.30, 95% CI (1.20, 1.40)], AST [SMD = -0.70, 95% CI (−1.28, −0.11)], LDH [SMD = −1.17, 95% CI (−1.37, −0.97], *p* < 0.00001], CK [SMD = −1.74, 95% CI (−2.34, −1.13)], TNF-α [SMD = −1.35, 95% CI (−1.85, −0.84)], and IL-6 [SMD = −1.40, 95% CI (−1.76, −1.05)]. There were no significant differences in the incidence of adverse reactions [RR = 1.56, 95% CI (0.73, 3.33), *p* = 0.25] or cTnI levels [SMD = −3.35, 95% CI (−6.81, 0.11)] between the groups.

**Conclusion:**

Shenmai injection with conventional treatment can reduce the degree of myocardial injury in patients with VMS, weaken the inflammatory response and improve the clinical efficacy of the conventional treatment. This approach was found to be safe. However, additional high-quality studies are necessary to confirm the reliability of this treatment method.

**Systematic Review Registration:**

https://www.crd.york.ac.uk/PROSPERO/logout.php, identifier PROSPERO (CRD42024518665).

## 1 Introduction

Viral myocarditis (VMC) refers to a nonspecific disease caused by infection with various cardiomyotropic viruses, which damage the myocardial structure and function. Coxsackievirus, orphan (Echo) virus, and poliovirus are among the most common cardiomyotropic viruses, among which Coxsackievirus is the most common pathogen and accounts for approximately 30%–50% of VMC cases. In recent years, reports have indicated that parvovirus B19 and human herpesvirus are common causes of VMS (Tavares et al., 2010). In addition, human adenovirus, influenza, rubella, herpes simplex, encephalitis, and hepatitis (A, B, C type) viruses, EB virus, cytomegalovirus, and human immunodeficiency virus (HIV) can cause myocarditis (Pollack et al., 2015; Lasrado and Reddy, 2020). The incidence of VMS is relatively high at approximately 11–20 per 100,000 people (Olejniczak et al., 2020). VMC is an important cause of heart failure and sudden death in young people. The pathogenesis of VMC has not been fully elucidated, but the recognized pathogenesis mainly includes ① direct action of the virus and ② combined action of the virus and the body’s immune response. The clinical manifestations of VMC are often related to the extent and location of the lesions. Patients with mild symptoms may experience only fatigue and shortness of breath, whereas those with severe symptoms may develop cardiogenic shock or even sudden death. Most patients may have prodromal symptoms of viral infection, such as fever, fatigue, sore throat, muscle aches and pains, or gastrointestinal symptoms, such as nausea and vomiting, 1–3 weeks before the onset of the disease. The main manifestations subsequently include cardiac pain, panic, dyspnea, edema, and even loss of consciousness and sudden death. Most patients with VMC complained of arrhythmia or had arrhythmia as the first symptom, and most of them had premature ventricular beats and conduction blocks. An increased heart rate may correspond to the degree of fever, and abnormal heart sounds or a gallop rhythm may be heard on auscultation. VMC is a self-limiting disease, and to date, it has no specific or effective treatment. Treatment often focuses on supportive therapy for left heart insufficiency. Appropriate rest, infection control, myocardial nourishment, antiviral treatment, free radical scavenging and other comprehensive therapeutic measures are recommended. Furthermore, symptomatic management of different clinical manifestations of VMC is performed.

The Shenmai injection is composed of the Chinese medicines *Panax ginseng* C. A. Mey [Araliaceae; *Ginseng radix et Rhizoma*] (Hong Shen) and *Ophiopogon japonicus* (Thunb.) Ker Gawl. [Asparagaceae; *O. japonicus radix*] (Mai Dong), which is included in the National Essential Drug List of China. Shenmai injection is derived from the TCM formula “Shen Mai Yin,” which was described in the text “Zheng Yin Mai Zhi.” This formula consists of the Chinese herbs Ren Shen and Mai Dong. In TCM, these herbs are believed to have the effects of “invigorating qi,” “solid doff,” and “nourishing yin.” The formula can be used to treat symptoms such as “fatigue” and “thirst,” which appear in the later stages of a fever (Dai et al., 2020). Shenmai injection is based on “Shen Mai Yin” but replaces Ren Shen with steamed Hong Shen. In TCM theory, the “invigorating qi” and “solid doff” effects of Hong Shen are stronger than those of regular ginseng, which is related to the unique ginsenosides produced during the hydrolysis and isomerization reactions that occur during the steaming process (Qu et al., 2023). Specifically, Hong Shen and Mai Dong were extracted by refluxing with ethanol; then, the filtrate was concentrated to obtain the red ginseng extract and maitake extract, which were subjected to crude filtration and ultrafiltration, respectively. The filtrate was further mixed, and polysorbate was added to dissolve it. After the solution had been rehydrated and the pH had been adjusted, Shenmai injection was performed, the main components of which were ginsenosides Rg1, Re, Rf, and Rb1. Previous studies have shown that the main active ingredient of Shenmai formula can reduce the inflammatory response in cardiovascular diseases by blocking the NF-κB pathway (Zhu et al., 2017). VMC commonly causes damage to cardiomyocytes, and studies have shown that Shenmai injection has various components that may improve myocardial injury through multiple targets and pathways (Zhang et al., 2021).

The Chinese medicine Hong Shen is the root of a perennial botanical drug of the family Wujiaceae. It is warm in nature, sweet in flavor and slightly bitter in taste and can tonify qi and blood, which can improve symptoms such as fatigue, insomnia and cough. In Chinese medicine, Mai Dong is the tuberous root on the collar root of leguminous plants (Liu and Wang, 2009). It is sweet, slightly bitter and slightly cold in nature. Traditional Chinese medicine (TCM) posits that sweetness can nourish yin, and coldness can clear heat, which nourishes yin, benefits the stomach, moistens the lung and generates fluids. Furthermore, Mai Dong can improve symptoms such as cough, dry throat and thirst. Pharmacological studies have shown that ginsenosides and a series of amino acids in Hong Shen can effectively improve the symptoms of myocardial ischemia and achieve cardiac strengthening. The results of high-performance liquid chromatography (HPLC) fingerprint peak identification reveal that the main active ingredients of Shenmai Injection include ginsenosides Rg1, Re, Rf, and Rb1 (Yang et al., 2023), of which ginsenosides Re and Rb3 can protect the myocardial cells of patients with VMC by reducing the level of peroxides in the body (Li et al., 2019). Mai Dong contains steroidal saponins and amino acids, which can actively improve the symptoms of cardiac arrhythmia. Shenmai injection can improve cardiac pumping, increase the cardiac output, reduce the peripheral vascular resistance, positively regulate the myocardial metabolism, and reduce symptoms of cardiac insufficiency. However, Shenmai injection can strengthen the function of mononuclear macrophages, increase the body’s ability to resist infection and improve the immune function.

## 2 Materials and methods

This meta-analysis was conducted in accordance with the Preferred Reporting Items for Systematic Reviews and Meta-Analysis (PRISMA) guidelines (Supplementary Material 1). The study was successfully registered on PROSPERO (CRD42024518665).

### 2.1 Database and search strategies

The PubMed, Cochrane Library, Web of Science, Excerpta Medica Database (Embase), China National Knowledge Infrastructure Database (CNKI), Wanfang Database (WF) and China Science and Technology Journal Database (VIP) databases were comprehensively searched from inception to 20 January 2024, with restricted languages of Chinese and English. In terms of search strategy, a combination of subject and free text terms was used, and different search strategies were applied to Chinese and foreign language databases. (Details of the search strategy are provided in Supplementary Table 1).

### 2.2 Inclusion criteria

The inclusion criteria for our study were constructed via the Population, Intervention, Control, and Outcome Study (PICOS) framework, which considers study design, participants, interventions, comparisons, and outcomes (Hutton et al., 2015). The specific inclusion criteria were as follows.

#### 2.2.1 Study design

Randomized controlled trials (RCTs) were conducted in China or other countries and published in Chinese or English.

#### 2.2.2 Participants

Patients were diagnosed with VMC regardless of age, sex, race, combined symptoms, or disease course.

#### 2.2.3 Interventions

The intervention consisted of a single administration of Shenmai injection for intravenous drip treatment alone or in combination with conventional treatment for VMC.

#### 2.2.4 Comparators

Control treatments were placebo or conventional treatment for VMC, which consisted of myocardial nourishment (e.g., coenzyme Q10, polarizing fluid, and inosine), antiviral agents, and immunomodulation agents.

#### 2.2.5 Outcomes

The primary outcomes were the clinical effectiveness rate and creatine kinase isoenzyme (CKMB) concentration. The secondary outcomes were cardiac troponin I (cTnI), lactate dehydrogenase (LDH), creatine kinase (CK), tumor necrosis factor alpha (TNF-α), interleukin-6 (IL-6), aspartate transaminase (AST), adverse reactions, and the effective rate of the electrocardiogram (ECG).

### 2.3 Exclusion criteria

Studies that satisfied any of the following criteria were excluded:

1. Systematic reviews or meta-analyses, observational studies, theoretical explorations, case reports, and animal or cell experiments.
2. Duplicate publications (in cases of duplicate studies, studies with the most recent and comprehensive data were selected).
3. Lack of randomized controlled trials.
4. Studies for which the full text could not be accessed online or via email.
5. Inappropriate intervention: The experimental group used other traditional Chinese medical treatments in addition to Shenmai injection therapy.
6. Low-quality studies such as those with flawed study designs or improper statistical methods.
7. Incomplete studies such as those that were not validated for clinical efficacy assessments.

### 2.4 Literature screening and data extraction

The literature selection was independently conducted by two assessors, who excluded irrelevant studies by screening the titles and abstracts. The full-text versions of these studies were downloaded and reviewed, and data were extracted and cross-checked. If there was a disagreement between the two researchers, a third senior researcher conducted a comprehensive evaluation. If data were missing from the article or could not be directly obtained, we attempted to contact the corresponding authors of the article to obtain the relevant complete data. The following data were extracted: 1) basic information, including the study name, first author’s name, publication year, and journal of publication; 2) baseline characteristics of the study subjects, including sample size, age, and sex; 3) intervention measures, including the dose of Shenmai injection, duration of intervention, and conventional treatment methods; 4) outcome indicators.

### 2.5 Risk of bias assessment

The methodological quality of each study was independently assessed by two reviewers via the Cochrane Risk of Bias 2 (RoB 2) tool (Higgins et al., 2023). Any discrepancies between the two reviewers were resolved via discussion with a third reviewer. The quality of the included RCTs was evaluated based on the following domains: 1) randomization process; 2) deviations from intended interventions; 3) missing outcome data; 4) measurement of the outcome; 5) selection of the reported result. The risk of bias of studies was classified into three levels: “high risk,” “low risk” and “unclear risk.”

### 2.6 Statistical analysis

This review used Review Manager 5.4 and Stata 16 for data analysis. Continuous variables were assessed by the standardized mean difference (SMD) and 95% confidence interval (95% CI), whereas categorical variables were measured by the risk ratio (RR) and 95% CI. *p* < 0.05 was considered to indicate statistical significance. An I^2^ ≤ 50% indicates low heterogeneity between studies, and a fixed effect model was selected for analysis. An I^2^ > 50% indicates significant heterogeneity between studies, and a random effects model was selected for analysis. Furthermore, the potential sources of heterogeneity were analyzed via subgroup and sensitivity analyses.

### 2.7 Subgroup analysis

If the results were heterogeneous, subgroup analyses were performed according to the treatment duration (≤3 weeks or >3 weeks) and dose (<50 mL/d or ≥50 mL/d) to explore sources of heterogeneity. Differences between subgroups were also analyzed.

### 2.8 Sensitivity analysis

When there was significant heterogeneity, sensitivity analyses were performed via the leave-one-out approach to assess the robustness of the findings and identify the sources of heterogeneity. These analyses were conducted via Review Manager 5.4.

### 2.9 Publication bias

When the number of included studies that examined a particular outcome exceeded 10, the publication bias was assessed by constructing a funnel plot with Review Manager 5.4 and performing Egger’s test via Stata 16.0 (Sterne et al., 2011).

### 2.10 Evidence confidence

The GRADE framework (Schünemann et al., 2020) was used to assess the quality of evidence for the included studies. The quality of evidence for the outcomes was assessed across five domains: study limitations, inconsistency, indirectness, imprecision, and publication bias.

## 3 Results

### 3.1 Search results

In total, 946 studies were initially identified from the literature search, and 592 duplicates were manually excluded through EndNote. After screening the titles and abstracts of the remaining 354 studies, 96 studies were excluded, including pharmacological research, animal experiments, cell experiments, systematic reviews, meta-analyses, summaries of experience, summaries and case reports. After screening the full texts of the remaining 258 studies, 240 articles were excluded for the following reasons: the full text was not available, outcome indicators were not reported, combination treatment with other botanical drug injections was used, combination treatment with other TCM therapies was used, information was lacking, and low-quality research was performed.

**FIGURE 1:**
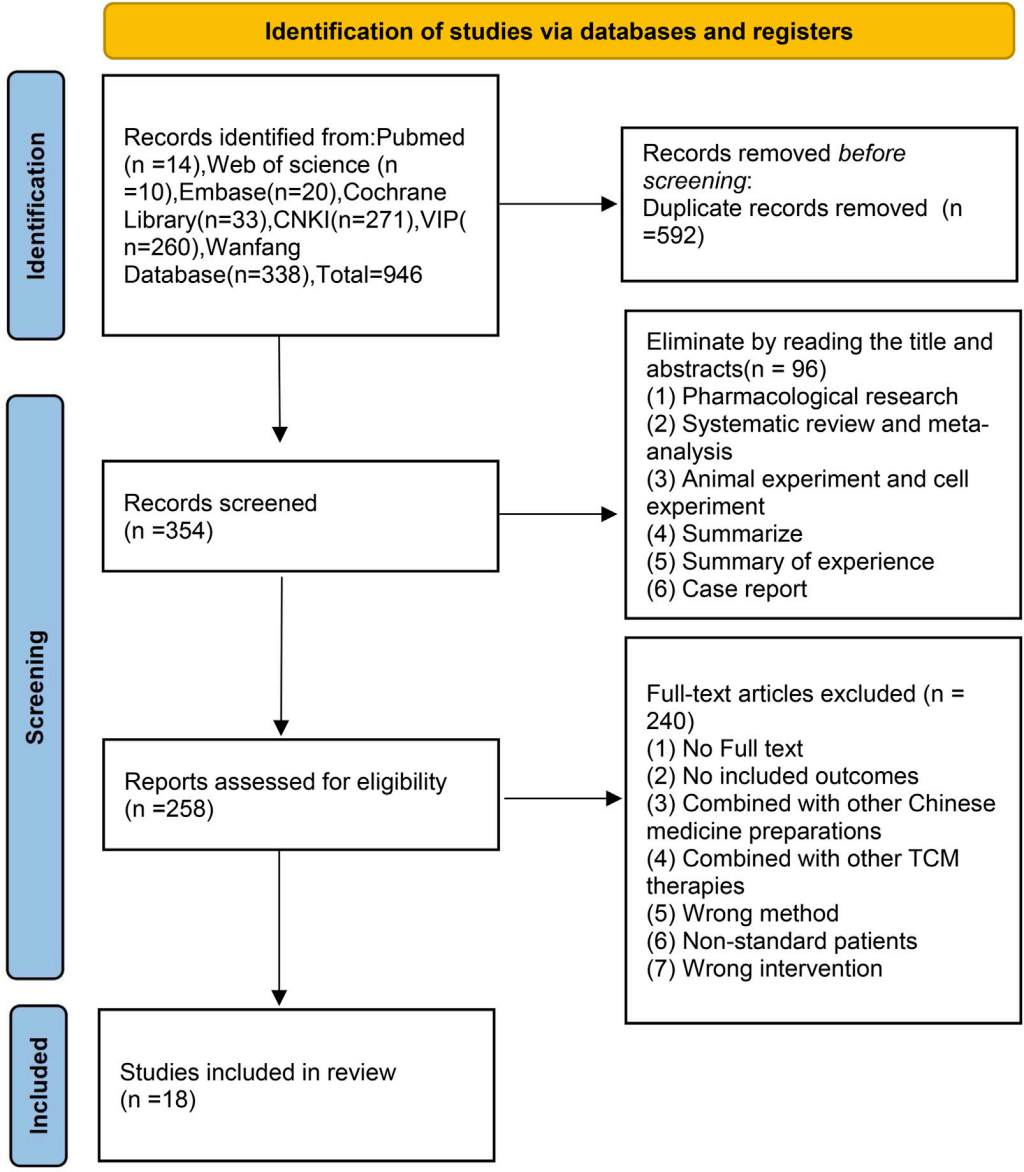
Literature screening process.

Ultimately, 18 studies (Song, 2006; Weng, 2006; Zhu et al., 2006; Chan and Liu, 2007; Hu et al., 2007; Wang et al., 2007; Hou, 2009; Liu, 2009; Lei, 2010; Xu, 2010; Bai, 2014; Xing et al., 2014; Luo et al., 2017; Lu et al., 2018; Shi and Xiang, 2018; Wei and Sun, 2019; Wan et al., 2020; Liang et al., 2022) were considered eligible for this systematic review. The research selection process is shown in Figure 1.

### 3.2 Study characteristics

The 18 included studies were published between 2006 and 2024, were conducted in China, included 1,661 patients with VMC, and were RCTs. The duration of the interventions was 14–30 days. The control groups were treated with conventional therapy, mostly antiviral therapy plus myocardial nutrition, and antiarrhythmic therapy for patients with abnormal ECGs. Song’s article stated the specific drugs and dosages, whereas the other studies listed only some of the drugs and did not state the dosages for the control treatments. The observation groups were treated with conventional therapy plus Shenmai injection or Shenmai injection combined with Western drugs. The dosages were 5–60 mL/d, and the frequency of use was once a day. The basic characteristics of the studies are shown in Table 1.

**TABLE 1:** Feature list.

Studies	Sample sizes	Gender (M/F)		Age(y)		Intervention		Duration	CT drugs	Outcomes
	(T/C)	T	C	T	C	T	C			
Liang et al. (2022)	76/76	38/38	40/36	53.3 ± 9.7	54.1 ± 10.2	SI, 40 mL, qd + CT	CT	2 weeks	Antiviral, nourishing myocardium, polarizing liquid	⑧⑨⑩
Wan et al. (2020)	70/70	38/32	36/34	55.1 ± 10.1	54.3 ± 9.5	SI, 60 mL, qd + CT	CT	2 weeks	Antiviral, vitamin C tablets, polarizing liquid, Coenzyme Q10 capsules, anti-arrhythmia	①②④⑧⑩
Lu et al. (2018)	43/43	21/22	19/24	47.34 ± 2.01	46.03 ± 1.97	SI, 40 mL, qd + CT	CT	2 weeks	Antiviral, immunity, nutrition, myocardium, anti-arrhythmia, Trimetazidine	③⑨
Shi and Xiang (2018)	49/49	25/24	28/21	34.4 ± 4.3	34.8 ± 4.2	SI, 60 mL, qd + CT	CT	30 days	Nourishing myocardium (coenzyme Q10, polarizing liquid, inosine), antiviral, immune regulation, dexamethasone sodium phosphate	①②⑤⑥⑦⑧⑩
Luo et al. (2017)	68/68	35/33	37/31	52.8 ± 9.2	54.1 ± 8.8	SI,60 mL, qd + CT	CT	2 weeks	Trimetazidine, L-carnitine	①⑧⑨
Xing et al. (2014)	82/82	91/73		2–14		SI, 60 mL, qd+ +CT	CT	15 days	Antiviral, antibacterial agent, cytochrome C, nutritional cardiometabolic drug, creatine phosphate sodium injection	①②③
Hou (2009)	62/60	30/32	28/32	38.2	38.4	SI, 40 mL, qd + CT	CT	15 days	Myocardial nutrition drugs, etc	①
Wang et al. (2007)	32/30	18/14	17/13	7.6	7.8	SI, 0.5 mL/kg-1 ml/kg, qd + CT	CT	15 days	Riboside, vitamin C, energy mixture, coenzyme Q10, antiarrhythmic drug, fructose 1,6 diphosphate	①②③
Chan and Liu (2007)	30/30	34/26		8–40		SI, 60 mL, qd + CT	CT	30 days	Coenzyme A, adenosine diphosphate	①
Weng (2006)	24/24	10/14	8/16	25.12 ± 12.51	26.12 ± 10.52	SI, 40 mL, qd + CT	CT	20 days	Antiviral, infection prevention, energy mixture, polarizing liquid, potassium magnesium aspartate needle	①
Zhu et al. (2006)	40/30	38/32		7.5 (2–13)		SI, 60 mL, qd + CT	CT	21 days	Ribavirin, vitamin C, energy mixture	①②⑥
Wei and Sun (2019)	48/49	25/23	26/23	(7.87 ± 1.22)	(7.82 ± 1.25)	Fructose 1,6 diphosphate, .0.25 g/kg, qd + SI, 50 mL, qd + CT	CT + Fructose 1,6 diphosphate,.0.25 g/kg, qd	2 weeks	—	①②⑥⑦
Bai (2014)	40/40	23/17	22/18	(52.8 ± 6.7)	(53.2 ± 7.2)	SI, 60 mL, qd + CT	CT	4 weeks	Vitamin C, energy injection, cyclic adenosine phosphate, antiviral therapy	①②④⑤⑥⑩
Xu (2010)	22/18	—		—		SI, 30 mL, qd + CT	CT	21 days	Antiviral therapy, vitamin C, coenzyme A, α-interferon	①
Lei (2010)	36/33	43/26		8.1 (2–15)		SI, 5–20 mL, qd + CT	CT	30 days	Energy mixture, vitamin C, inosine, cytochrome C, antiviral therapy	①②③⑤⑥⑦
Liu (2009)	60/60	33/27	38/22	6–13		SI, 1–1.5 mL/kg, qd + CT	CT	28 days	Symptomatic treatment, nutritional support treatment, sodium fructose diphosphonate	①③
Hu et al. (2007)	25/24	31/18		7.5 (2–14)		SI, 5–20 mL, qd + CT	CT	30 days	Energy mixture, vitamin C, inosine, cytochrome C	①②⑤⑥⑦⑩
Song (2006)	52/52	29/23	26/26	31.4	30.2	SI, 20 mL, qd + CT	CT	3 weeks	Coenzyme A, 100 u, qd + ATP, 40 mg, qd + vitamin C,4 g, qd	①③

T, treatment group; C, control group; SI, shenmai injection; NM, not mentioned; w, week, d, day; tid, three times a day; qd, once a day; CT, conventional treatment. ① Total efficacy rate. ② CKMB. ③ Electrocardiogram efficacy rate. ④ cTnI, ⑤ AST, ⑥ LDH, ⑦ CK, ⑧ TNF-α, ⑨ IL-6, and ⑩ adverse reactions.

### 3.3 Quality assessment of the included studies

**FIGURE 2:**
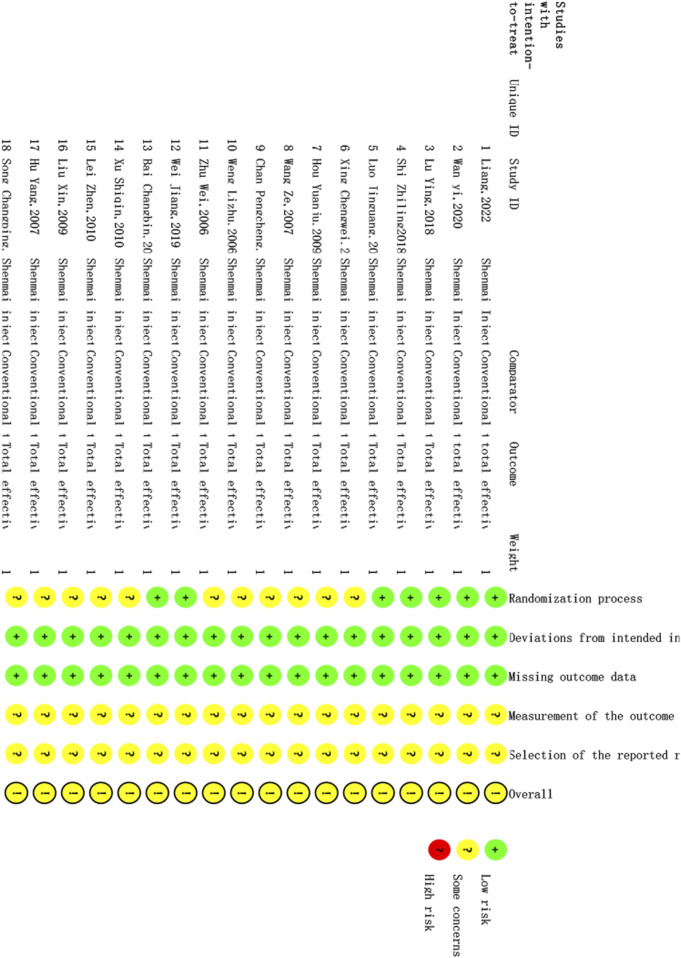
Quality assessment.

The quality of the 18 studies included was evaluated via RoB 2. The results of the ROB evaluation are shown in Figure 2. Seven randomized controlled trials described the use of the random number table method; thus, there was a low risk of bias regarding the randomization process. Eleven randomized controlled trials mentioned randomization without specifying the details of the generation of random sequences; therefore, there was an unclear risk of bias regarding the randomization process. Some of the randomized controlled trials in this article reported the use of blinding, but whether blinding was specifically used for patients, experimenters, and evaluators or only for patients was not described in detail; however, no patients were reassigned during the trial, and the researchers used appropriate statistical methods. Therefore, there was a low risk of bias regarding deviations from the intended intervention. The outcome data of all randomized controlled trials were complete with no reported missing outcome data; therefore, there was a low risk of bias regarding missing outcome data. There were no reports of blinding of the outcome assessors in randomized controlled trials, and it is unclear whether the intervention’s outcome was affected; however, any such effect is likely small, and there was an unclear risk of bias regarding the outcome assessment process. Prespecified statistical plans were not available; therefore, it cannot be confirmed whether the statistical methods were consistent with the prespecified methods. However, there are no reports of different evaluation methods for the outcome, and there are no reports of the use of other analysis methods. Thus, the risk of bias regarding selective reporting bias is uncertain.

### 3.4 Meta-analysis results

#### 3.4.1 Primary outcomes

##### 3.4.1.1 Meta-analysis of the total effectiveness rate

**FIGURE 3:**
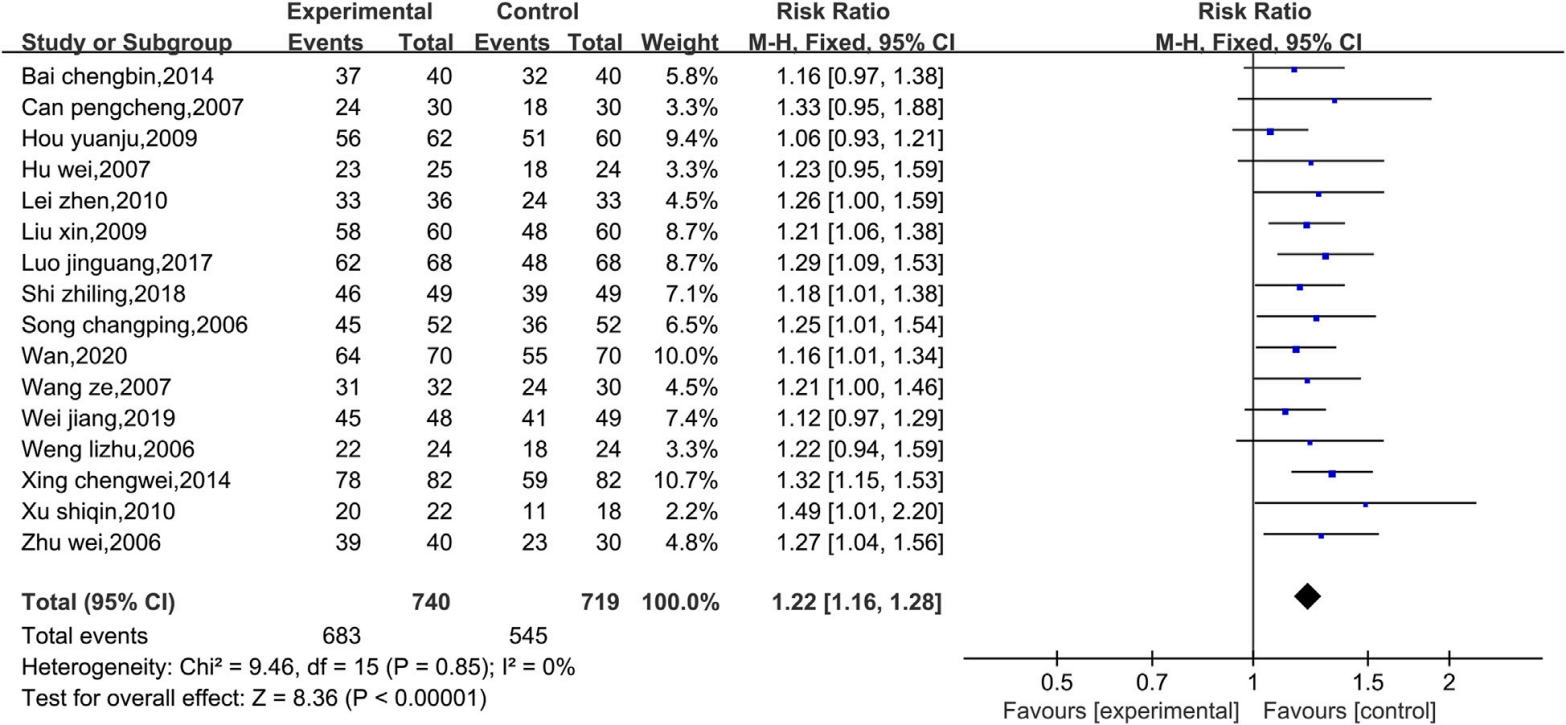
Comparative forest plots of the total efficacy rates.

Data on the total effectiveness rates were available in 16 studies, as shown in Figure 3. Due to the low level of heterogeneity (*p* = 0.85, I^2^ = 0%), a fixed effect model was used. Compared with that of the control group, the overall clinical efficacy of the experimental group was significantly greater [RR = 1.22, 95% CI (1.16, 1.28), *p* < 0.00001]. Therefore, compared with conventional Western medicine treatment, the combined use of Shenmai injection and conventional Western medicine treatment showed better clinical efficacy. Due to the low level of heterogeneity, subgroup and sensitivity analyses were not conducted.

##### 3.4.1.2 Meta-analysis of CKMB

**FIGURE 4:**
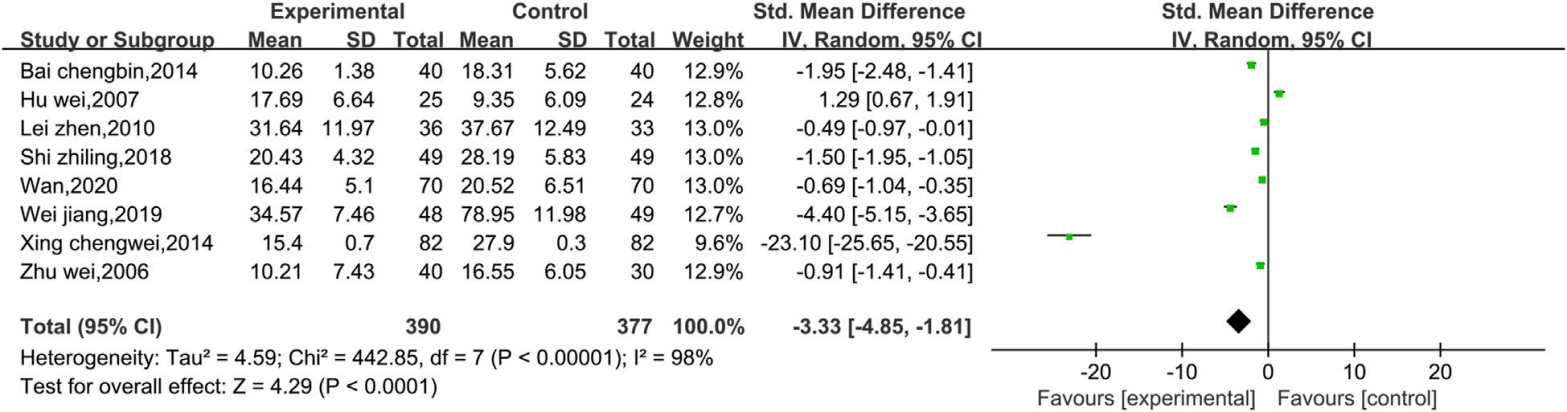
Comparative forest plots of the CKMB levels.

**FIGURE 5:**
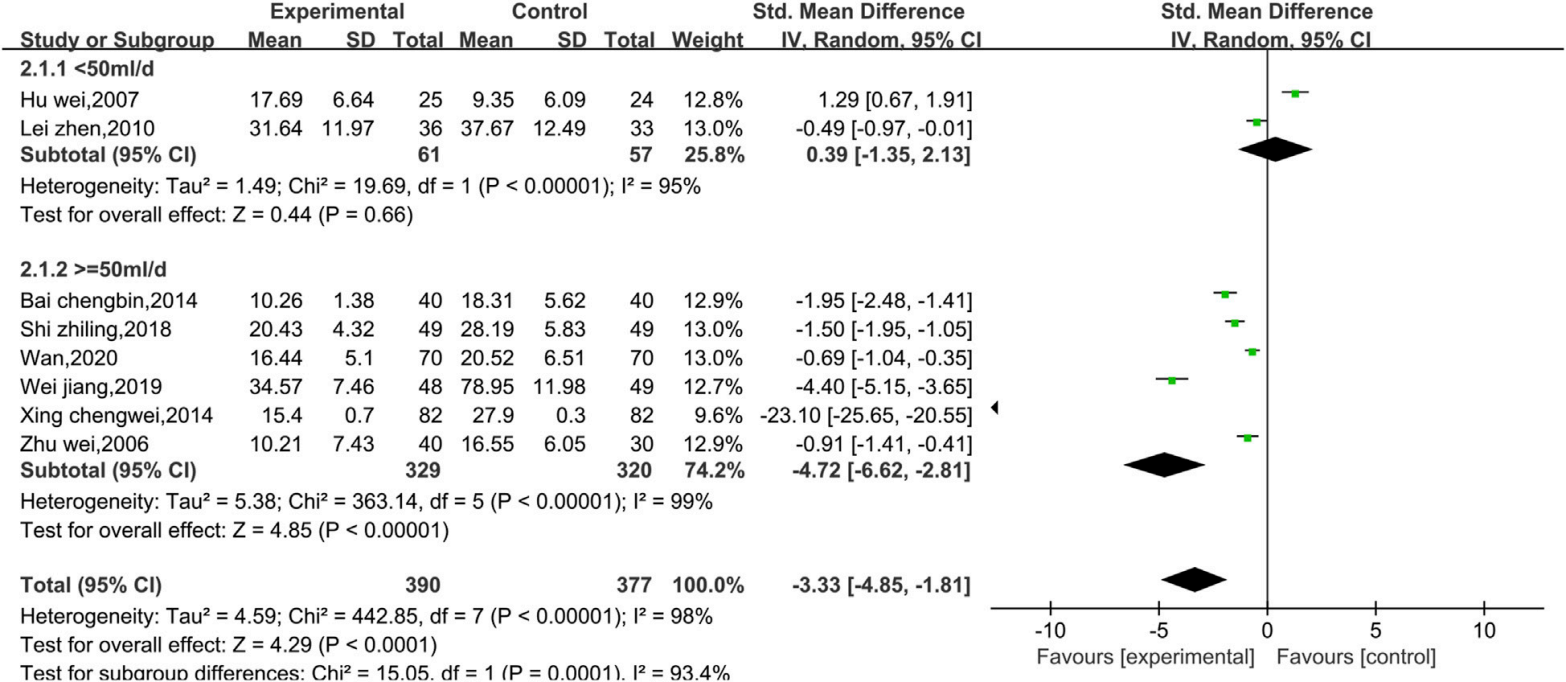
Forest plots of the subgroup analysis at different doses (CKMB level).

**FIGURE 6:**
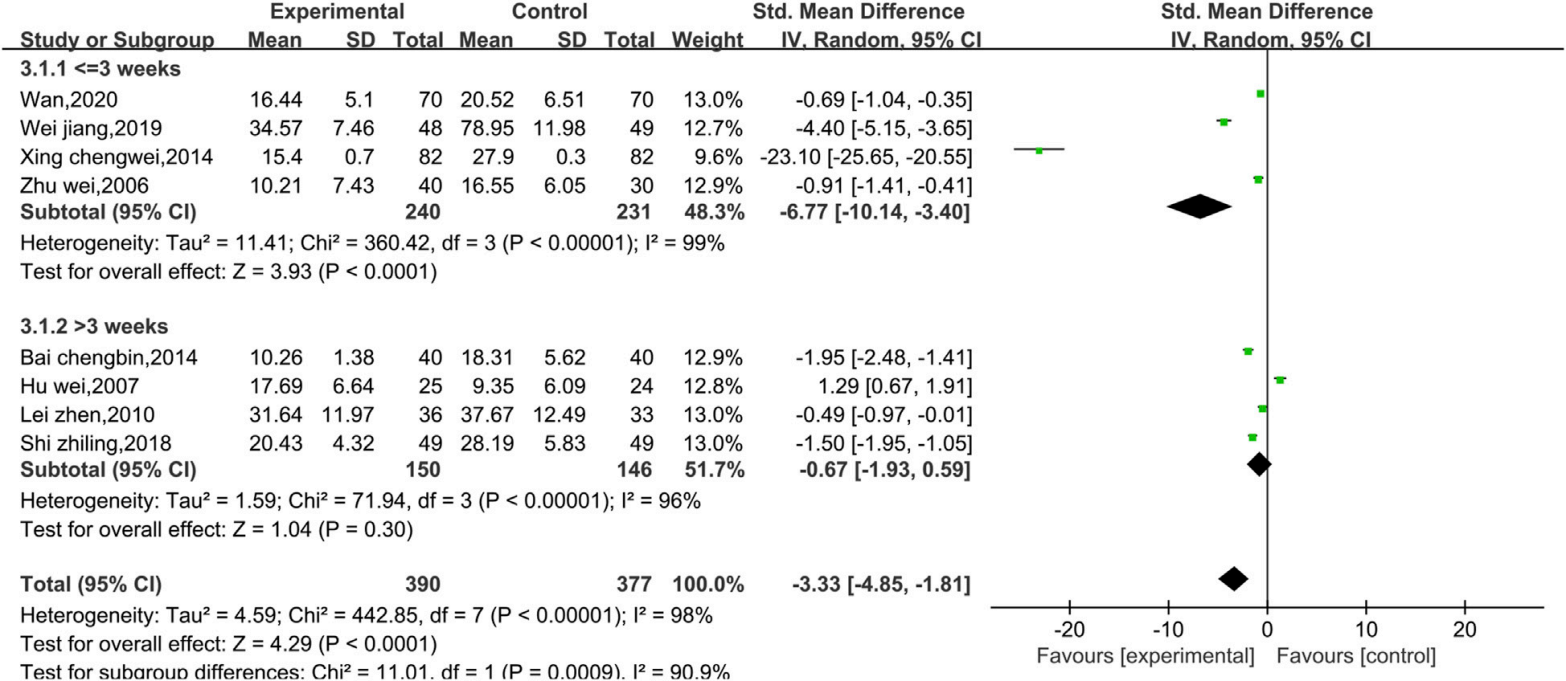
Forest plots of the subgroup analysis at different durations of treatment (CKMB level).

Eight studies reported data on CKMB, as shown in Figure 4. Due to the high level of heterogeneity (*p* < 0.00001, I^2^ = 98%), a random effect model was used. The results show that the combination of Shenmai injection with conventional Western medicine could better reduce CKMB in patients than the conventional Western medicine therapy alone [SMD = −3.33, 95% CI (−4.85, −1.81), *p* < 0.0001]. Subgroup analyses were performed based on differences in therapeutic dose and duration of treatment (Figure 5). Patients were further divided into two dosage subgroups: <50 mL/d (*p* < 0.00001, I^2^ = 95%) and ≥50 mL/d (*p* < 0.00001, I^2^ = 99%). The outcomes were significantly different between these dosage subgroups (χ^2^ = 15.05, df = 1, *p* = 0.0001), which indicates that there was a greater decrease in CKMB after treatment with high-dose Shenmai injection. Patients were also divided into two subgroups based on the duration of continuous treatment (Figure 6): ≤3 weeks (*p* < 0.00001, I^2^ = 99%) and >3 weeks (*p* < 0.00001, I^2^ = 96%). The outcomes were significantly different between these treatment duration subgroups (χ^2^ = 11.01, df = 1, *p* = 0.0009), which indicates that a treatment duration of 3 weeks or less was more beneficial for reducing CKMB. Sensitivity analyses were performed using the leave-one-out method; the pooled results were robust.

#### 3.4.2 Secondary outcomes

##### 3.4.2.1 Meta-analysis of the efficacy rate of ECG

**FIGURE 7:**
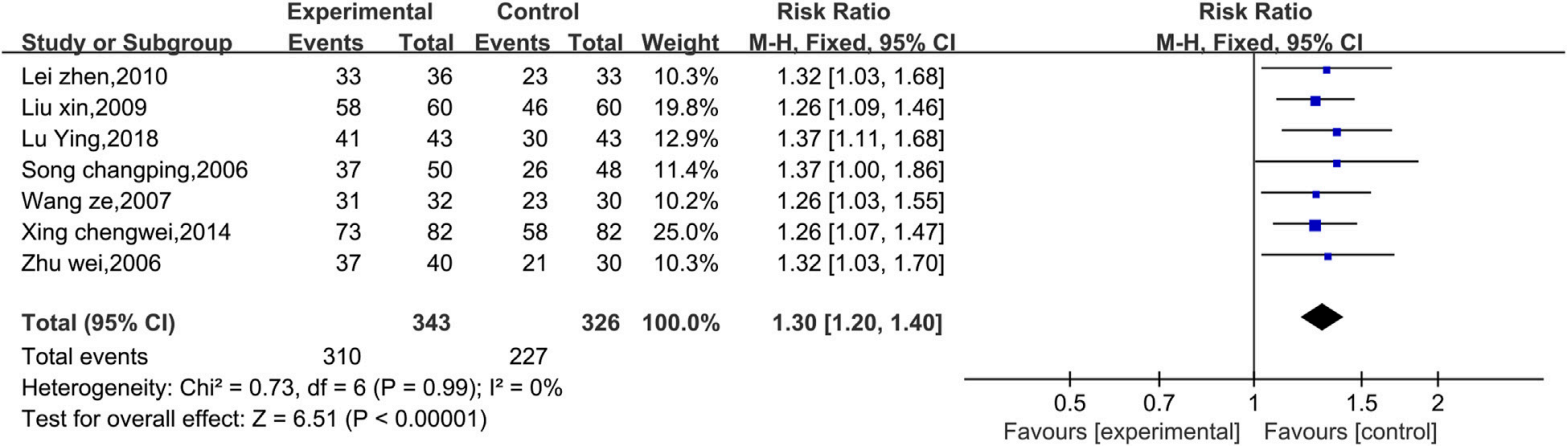
Comparative forest plots of the efficacy rates of electrocardiography.

Seven studies reported preliminary data on the ECG efficacy (Figure 7). Due to the low level of heterogeneity (*p* = 0.99, I^2^ = 0%), a fixed effect model was used. The results of the analysis revealed that the ECG efficacy of the experimental group was significantly greater than that of the control group [RR = 1.30, 95% CI (1.20, 1.40), *p* < 0.00001]. Therefore, the combination of Shenmai injection samples with conventional Western drug therapy better improved the ECG results among VMC patients than conventional Western drug therapy. Subgroup and sensitivity analyses were not performed because the low level of heterogeneity was less than 50%.

##### 3.4.2.2 Meta-analysis of cTnI

**FIGURE 8:**
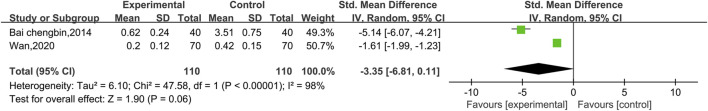
Comparative forest plots of the cTnI levels.

**FIGURE 9:**
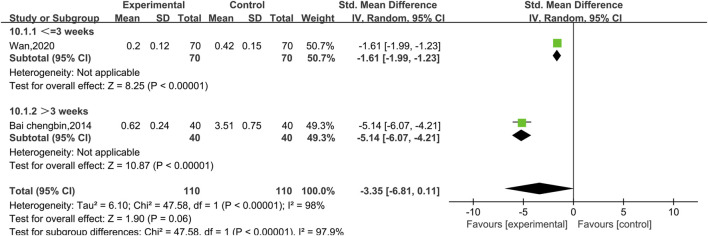
Forest plots of the subgroup analysis at different durations of treatment (cTnI level).

Two experiments reported data on cTnI, as shown in Figure 8. There was a high level of heterogeneity (*p* < 0.00001, I^2^ = 98%); therefore, a random effect model was used. The results reveal no significant difference between conventional treatment with Western medicine and treatment with Shenmai injection in combination with Western medicine in terms of reducing the cTnI index of patients [SMD = −3.35, 95% CI (−6.81, 0.11), *p* = 0.06]. Due to the high heterogeneity, subgroup analyses were performed; however, because there was only one study in each subgroup, the I^2^ value could not be calculated. The treatment dose was identical in both trials, so subgroup analysis was performed based on the treatment duration (Figure 9). There was a significant difference between the subgroups (χ^2^ = 47.58, df = 1, *p* < 0.00001), which indicates that a longer treatment duration of Shenmai injection may be more favorable for lowering cTnI in patients. However, too few randomized controlled trials were included in this analysis, which leads to a high degree of heterogeneity.

##### 3.4.2.3 Meta-analysis of ASTs

**FIGURE 10:**
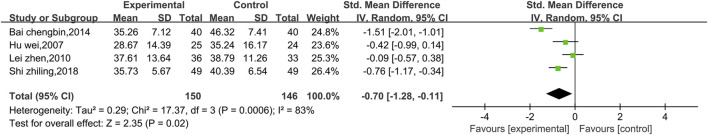
Comparative forest plots of the AST levels.

**FIGURE 11:**
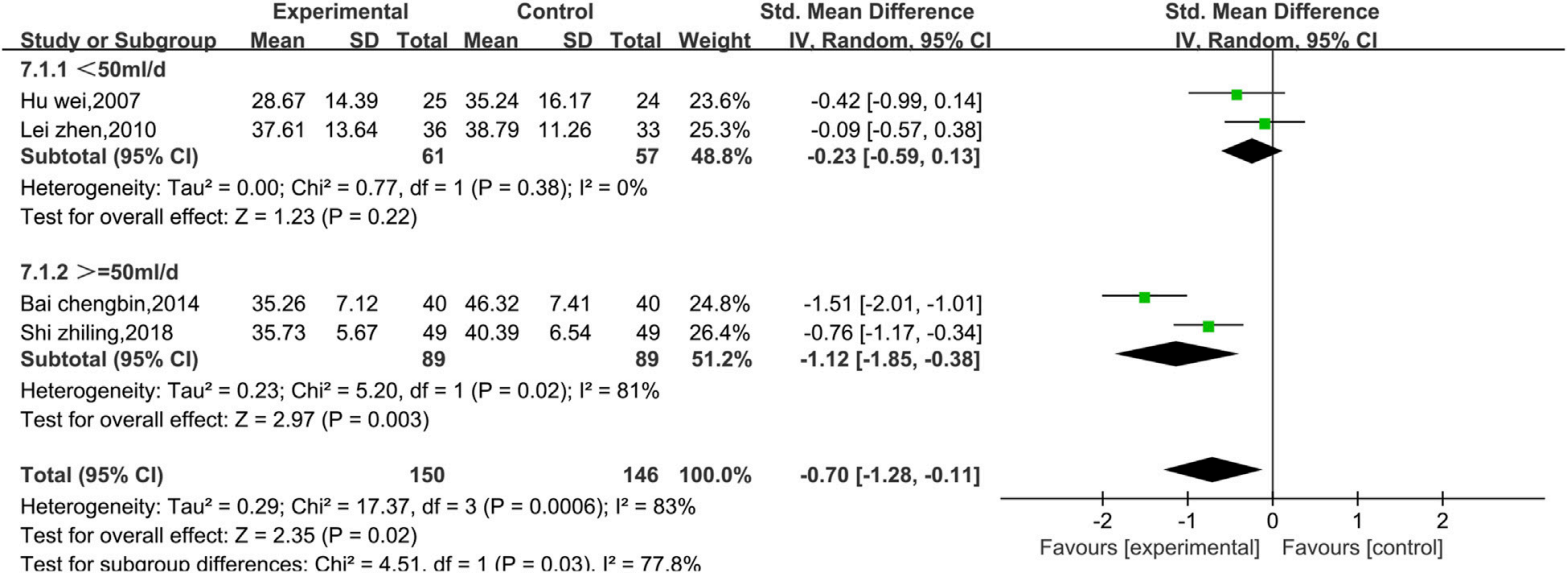
Forest plots of the subgroup analysis at different doses (AST level).

Four studies reported AST data, as shown in Figure 10. There was a high level of heterogeneity (*p* = 0.0006, I^2^ = 83%); thus, a random effects model was used. The results show that the combination of Shenmai injection with conventional Western medicine could better reduce the AST levels in patients than conventional Western medicine therapy alone [SMD = −0.70, 95% CI (−1.28, −0.11), *p* = 0.02]. At I^2^>50%, the treatment duration was identical in all four experiments; therefore, we performed subgroup analysis only based on the treatment dosage. The studies were divided into a low-dosage group (χ^2^ = 0.77, I^2^ = 0%) and a high-dosage group (χ^2^ = 5.20, I^2^ = 81%). The results (Figure 11) reveal a significant difference between the subgroups (χ^2^ = 4.51, df = 1, *p* = 0.03), which indicates that Shenmai injection at a dosage of 50 mL/d or higher led to a more pronounced reduction in AST levels among VMC patients. Sensitivity analysis was performed using the leave-one-out approach, and the pooled results were shown to be robust.

##### 3.4.2.4 Meta-analysis of LDH

**FIGURE 12:**
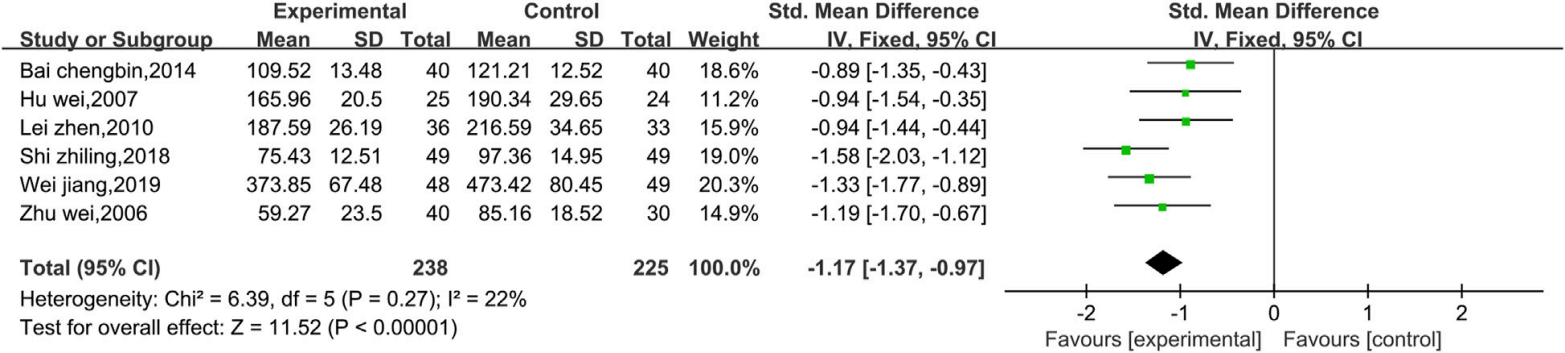
Comparative forest plots of the LDH levels.

Six studies reported data on LDH (Figure 12). There was a low level of heterogeneity (*p* = 0.27, I^2^ = 22%); thus, a fixed effect model was used. The meta-analysis reveals that Shenmai injection in combination with conventional treatment could better reduce LDH in patients than the conventional treatment alone [SMD = −1.17, 95% CI (−1.37, 0.97), *p* < 0.00001]. Subgroup and sensitivity analyses were not performed because of the low level of heterogeneity.

##### 3.4.2.5 Meta-analysis of CK

**FIGURE 13:**
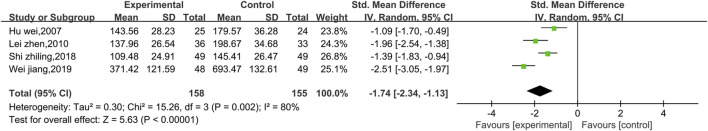
Comparative forest plots of the CK levels.

**FIGURE 14:**
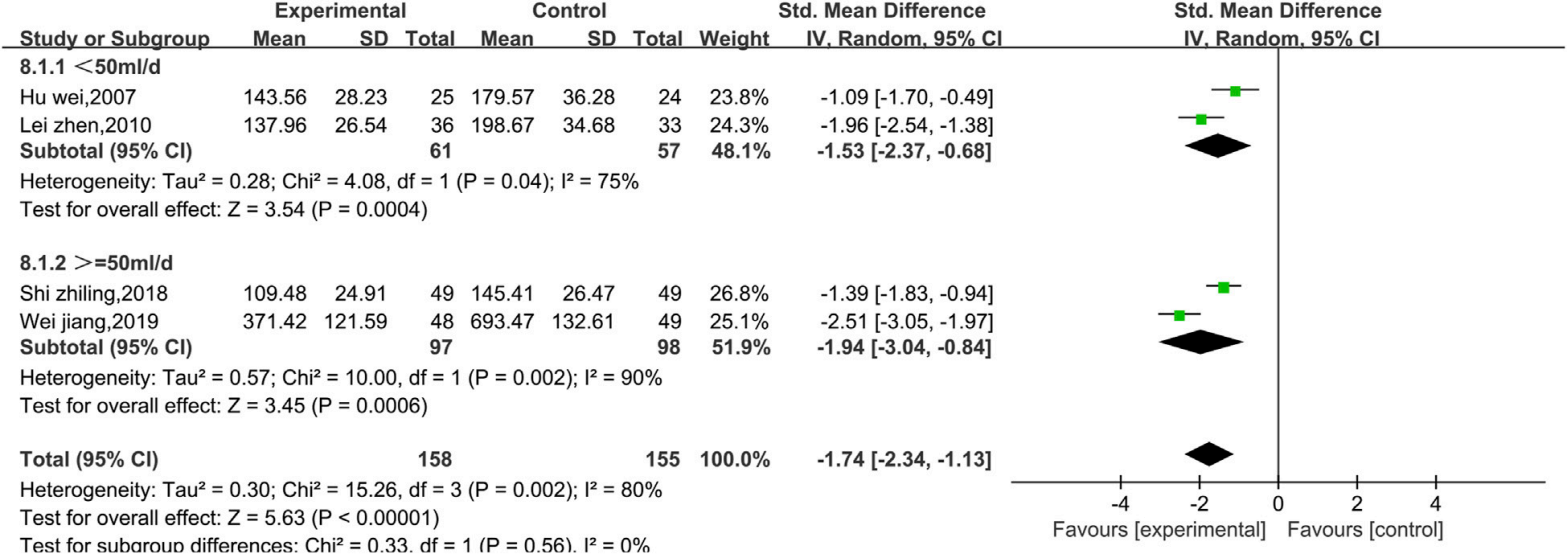
Forest plots of the subgroup analysis at different doses (CK level).

**FIGURE 15:**
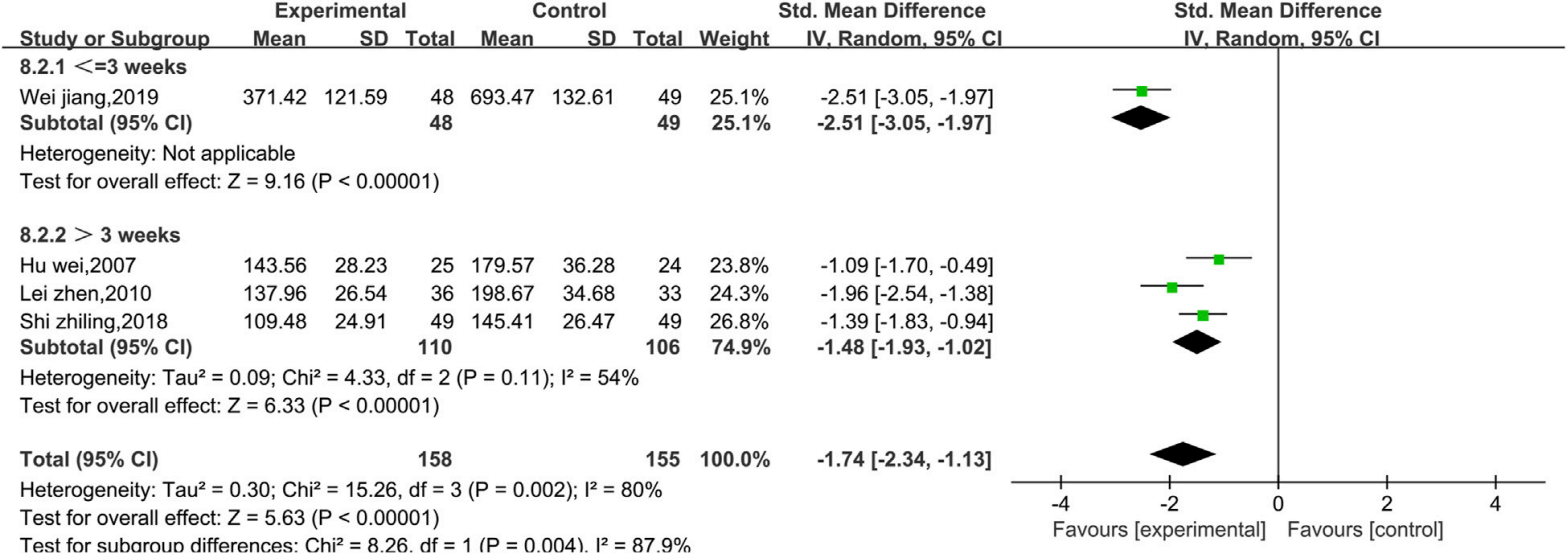
Forest plots of the subgroup analysis at different durations of treatment (CK level).

Four studies reported data on CK (Figure 13). There was a high level of heterogeneity (*p* = 0.002, I^2^ = 80%); thus, a random effect model was used. The meta-analysis reveals that the CK index more significantly decreased in patients treated with the combination of Shenmai injection and conventional Western medicine than in those treated with conventional therapy [SMD = −1.74, 95% CI (−2.34, −1.13), *p* < 0.00001]. Due to I^2^>50%, subgroup analysis was conducted based on the treatment dosage (Figure 14) and treatment duration (Figure 15). There were no significant differences between the low-dosage subgroup (χ^2^ = 4.08, I^2^ = 75%) and the high-dosage subgroup (χ^2^ = 10.00, I^2^ = 90%) (χ^2^ = 0.33, df = 1. *p* = 0.56). With respect to treatment duration, there was only one study in the <3-week subgroup; therefore, no I^2^ value could be calculated. There were 3 studies in the >3-week subgroup (χ^2^ = 4.33, I^2^ = 54%). Subgroup analysis reveals significant differences between the treatment duration subgroups (χ^2^ = 8.26, df = 1, *p* = 0.004), which indicates that the treatment with Shenmai injection for 3 weeks or fewer led to a more pronounced reduction in CK among VMC patients. Sensitivity analysis was performed using the leave-one-out approach, and the pooled results were shown to be robust.

##### 3.4.2.6 Meta-analysis of TNF-α

Four studies reported data on TNF-α, as shown in Supplementary Figure 1. There was a high level of heterogeneity (*p* = 0.0001, I^2^ = 85%); thus, a random effect model was used. The results of the meta-analysis revealed that the combination of Shenmai injection with conventional treatment was more beneficial to the recovery of TNF-thin patients, since the TNF-y of TNF was lower after Shenmai injection treatment than after the conventional treatment alone [SMD = −1.35, 95% CI (−1.85, −0.84), *p* < 0.00001]. I^2^ > 50%, and subgroup analyses were performed based on the therapeutic dosage (Supplementary Figure 2). There was only one study in the low-dosage group, which prevented the calculation of an I^2^ value. There were three studies in the high-dosage subgroup (χ^2^ = 20.41, I^2^ = 90%). There was no significant difference between the treatment dosage subgroups (χ^2^ = 0.00, df = 1, *p* = 0.99). With respect to the treatment duration (Supplementary Figure 3), there was only one study in the >3-week subgroup, which prevented the calculation of an I^2^ value. There was no significant difference between the > 3-week subgroup and the < 3-week subgroup (χ^2^ = 15.71, I^2^ = 87%) (χ^2^ = 2.04, df = 1, *p* = 0.15). Sensitivity analysis was performed using the leave-one-out method and revealed that the level of heterogeneity was significantly reduced (I^2^ = 12%) when the study by Wan et al. (2020) was removed. The heterogeneity may have been due to differences in interventions, as no included trials specified the use of specific conventional treatments. No other sources of clinical heterogeneity were identified. After excluding this RCT, the meta-analysis was performed again via a fixed effects model (Supplementary Figure 4). The results reveal that the addition of Shenmai injection to conventional treatment was more favorable for reducing TNF-α levels in patients [SMD = −1.56, 95% CI (−1.78, −1.33), *p* < 0.00001].

##### 3.4.2.7 Meta-analysis of IL-6

Three studies reported data on IL-6, as shown in Supplementary Figure 5. There was a high level of heterogeneity (*p* = 0.09, I^2^ = 58%); thus, a random effects model was used. The meta-analysis reveals that the combination of Shenmai injection with conventional treatment more significantly reduced the IL-6 index of patients than the conventional treatment [SMD = −1.40, 95% CI (−1.76, −1.05), *p* < 0.00001]. With I^2^ >50%, subgroup analysis was performed based on the treatment dosage but not the treatment duration (the same treatment duration was used in all three articles) (Supplementary Figure 6). There was only one in the high-dosage subgroup, which prevented the calculation of an I^2^ value. There was also a low-dosage subgroup (χ^2^ = 4.17, I^2^ = 76%). The subgroup analysis reveals no significant differences between the treatment dosage subgroups (χ^2^ = 0.54, df = 1, *p* = 0.46). The sensitivity analysis reveals a significant reduction in heterogeneity (I^2^ = 0%) when the study of Lu et al. (2018) was excluded. The heterogeneity may have been due to the sample size of this study, which was significantly smaller than that in the other two studies. After excluding this RCT, the meta-analysis was performed again via a fixed effect model (Supplementary Figure 7). The results reveal that the addition of Shenmai injection to the conventional treatment better decreased the patients’ IL-6 levels [SMD = −1.24, 95% CI (−1.49, −0.98), *p* < 0.00001].

##### 3.4.2.8 Meta-analysis of adverse reactions

Nine of the included studies mentioned information about adverse reactions, four of which reported no adverse reactions during the treatment phase, and the remaining five studies reported the presence of adverse events during the treatment phase. Among them, 16 patients in the Shenmai injection treatment group experienced adverse reactions during treatment, including 4 cases of nausea and vomiting, 3 cases of headache, 2 cases of dizziness, 1 case of rash, 1 case of abdominal discomfort, 1 case of delirium, and 1 case of insomnia. In total, 10 patients in the conventional treatment group experienced adverse reactions during treatment, including 6 patients with headache, 3 patients with nausea and vomiting, and 1 patient with delirium. No significant heterogeneity was observed (I^2^ = 0.%, *p* = 0.87). Meta-analysis was performed via the fixed effects model (Supplementary Figure 8), and the results reveal that there was no significant difference between Shenmai injection group and conventional treatment group [RR = 1.56, 95% CI (0.73, 3.33), *p* = 0.25]. Subgroup and sensitivity analyses were not performed because of the low level of heterogeneity.

### 3.5 Sensitivity analysis

When there was significant heterogeneity, we used sensitivity analyses, including CKMB, AST, CK, TNF-α, and IL-6. Sensitivity analyses were performed by deleting articles one at a time. The level of heterogeneity appeared to change after the articles by Wan et al. (2020) or Lu et al. (2018) were excluded. The sensitivity analysis results are shown in Table 2.

**TABLE 2:** Sensitivity analysis.

Outcome	Study removed [first author (year)]	*p*-value, I^2^ value(%)	*p*-value	RR/SMD [95% CI]
TNF-α	Liang et al. (2022)	*p* < 0.0001, I^2^ = 90	*p* = 0.003	−1.35 [−2.08, −0.62]
	Luo et al. (2017)	*p* = 0.0004, I^2^ = 87	*p* < 0.0001	−1.24 [−1.87, −0.62]
	Shi and Xiang (2018)	*p* = 0.0004, I^2^ = 87	*p* < 0.0001	−1.22 [−1.80, −0.63]
	Wan et al. (2020)	*p* = 0.32, I^2^ = 12	*p* < 0.00001	−1.56 [−1.80, −1.32]
IL-6	Liang et al. (2022)	*p* = 0.05, I^2^ = 73	*p* < 0.00001	−1.53 [−2.14, −0.92]
	Luo et al. (2017)	*p* = 0.04, I^2^ = 76	*p* < 0.00001	−1.52 [−2.15, −0.89]
	Lu et al. (2018)	*p* = 0.94, I^2^ = 0	*p* < 0.00001	−1.24 [−1.49, −0.98]

### 3.6 Publication bias

We detected publication bias in the total efficacy rate, since ≥10 studies reported this outcome. The results are shown in Supplementary Figure 9. The effect points of each trial were symmetrically distributed in an inverted funnel shape, which indicates that there was a low risk of publication bias. However, this analysis was not sufficiently precise. Therefore, we used Stata 16.0 to perform Egger’s test; the results show that *p* = 0.1081, which is >0.05 and suggests that there was no significant publication bias.

### 3.7 Quality of evidence

The included studies did not provide detailed descriptions of allocation concealment or blinding specifics; therefore, all evidence was downgraded by one level due to limitations in the studies. There was significant heterogeneity for the following outcomes: CKMB, AST, CK, TNF-α, IL-6, and cTnI. Therefore, the evidence for these outcomes was downgraded by two grades in the inconsistency aspects. All endpoints could be used as direct evidence, so indirectness was not downgraded. AST, CK, IL-6, and cTnI were downgraded by one grade because the sample size was <400. Regarding adverse reactions, although they satisfied the OIS criteria, the evidence was downgraded by one grade for imprecision because the corresponding forest plots showed that the confidence intervals contained null values, and the credible intervals did not exclude significant benefit or harm. There was no risk of publication bias in terms of the effective rates; therefore, this evidence was not downgraded. Overall efficiency, ECG efficiency, and LDH were rated as “moderate”-quality evidence; adverse effects were rated as “low”-quality evidence; CKMB, AST, CK, TNF-α, IL-6, and cTnI were rated as “very low”-quality evidence; CKMB, AST, CK, TNF-α, IL-6, and cTnI were rated as “very low”-quality evidence. Supplementary Figure 10 shows the evidence quality analysis of the continuous variables. Supplementary Figure 11 shows the evidence quality analysis of the bicategorical variables.

## 4 Discussion

In total, 18 randomized controlled trials were included in this meta-analysis. The results indicate that compared to conventional treatment, the combination of Shenmai injection with conventional treatment more effectively improved the clinical efficacy of VMC treatment by improving the ECG findings of the patients and decreasing the physicochemical indices of the patients, including CKMB, AST, LDH, CK, TNF-α, and IL-6.

In cases of high heterogeneity, sensitivity analyses were performed by excluding individual studies one at a time and reconducting the meta-analyses. The results indicate that differences in the control group interventions and sample sizes of the studies can be sources of heterogeneity. This heterogeneity was reduced by excluding two experiments that were potential sources of heterogeneity. To assess the clinical heterogeneity, we performed subgroup analyses based on the treatment dosage and duration; we found that neither factor was a source of heterogeneity.

The clinical features of acute VMC include chest pain, fever, flu-like symptoms, gastrointestinal or respiratory symptoms and dyspnea. Some patients also experience syncope. Most patients with acute VMC experience ECG changes, the most common of which include similar ST-segment elevation to that in acute myocardial infarction, QRS width >120 ms, and arrhythmias. The gold standard for the diagnosis of myocarditis is endocardial myocardial biopsy and cardiac magnetic resonance imaging (Sayegh et al., 2023). However, laboratory tests are recommended when endocardial myocardial biopsy and cardiac magnetic resonance imaging are not feasible or when the patient’s status is unstable. Recommended laboratory tests include biomarkers of myocardial necrosis (e.g., CKMB, CK, cTnI), inflammatory markers (e.g., C-reactive protein, TNF-α, and IL-6) (Ammirati et al., 2020), and the myocardial enzymes serum LDH and aspartate transferase (AST), which may also be used as secondary evidence to diagnose VMS. Therefore, these endpoints were selected to assess the therapeutic effect in this study and provide a reference basis for the efficacy of Shenmai injection in combination with conventional modalities in the treatment of VMS.

Shenmai injection is made from the extracts of Hong Shen and Mai Dong. Hong Shen is composed of fresh Ren Shen; during the concoction process, ginsenosides undergo decarboxylation, hydrolysis and isomerization and result in new metabolites with stronger activity, such as ginsenosides Rg3, Rg5 and Rg6, which are unique to Hong Shen (Chu et al., 2013). These metabolites have been shown to have antiviral effects (Li et al., 2019), enhance body immunity (Zhao et al., 2014) and exhibit other effects. Their related preparations are mostly used to treat VMS. The main bioactive metabolites of maitake are steroidal saponins and flavonoids. Studies have shown that its metabolites have immunomodulatory, antiviral, and anti-inflammatory effects and protect the cardiovascular system (Lei et al., 2021)

The E3 ubiquitin ligase family members TRIM18 and TRIM29 may be involved in the development of viral myocarditis and can be therapeutic targets for viral myocarditis. TRIM18 recruits PPM1A, interacts with TANK-binding kinase 1 (TBK1), dephosphorylates and inactivates TBK1, blocks the interaction between TBK1 and its upstream mitochondrial antiviral signaling protein (MAVS) and STING, and inhibits type-I IFN-mediated antiviral signaling during viral infection. The knockdown of TRIM18 effectively protects the model mice from viral myocarditis, which further illustrates its potential as a therapeutic target. TRIM29 promotes cardiomyocyte apoptosis through the activation of the ER kinase (PKR)-like ER kinase (PERK)/activating transcription factor 4 (ATF4)/CHOP pathway, exacerbates myocardial damage caused by cardiomyopathic viruses and promotes the cardiomyopathic virus replication (Fang et al., 2022; Wang et al., 2024). However, the specific molecular mechanism of Shenmai injection in the treatment of viral myocarditis remains unclear. Shenmai injection may inhibit the expression of the autophagy-related genes Beclin-1 and LC3-II and apoptosis-related genes in viral myocarditis, inhibit excessive autophagy and apoptosis of cardiomyocytes in viral myocarditis, and subsequently inhibit the replication and release of viruses (Liang et al., 2022). Whether Shenmai injection can reduce the apoptosis of cardiomyocytes in viral myocarditis by inhibiting TRIM29 requires further research. Studies have also revealed that Shenmai injection may alleviate myocardial injury in viral myocarditis by lowering the expression levels of the NLRP3 inflammasome in peripheral blood mononuclear cells, which inhibits the cascading amplification of downstream inflammatory pathways (Wan et al., 2020). Currently, no reports indicate that Shenmai injection can improve viral myocarditis by inhibiting TRIM18 and TRIM29. TRIM18 and TRIM29 may be potential research directions for the treatment of viral myocarditis with Shenmai injection and herbal formulations.

According to the spontaneous reporting system (SRS) safety data analysis of Shenmai injection, among all adverse drug reaction (ADR) reports in 2005–2012, the adverse reactions associated with Shenmai injection mainly involved the dermatological system, gastrointestinal system, respiratory system, and systemic damage. The top 10 ADR manifestations were breathlessness, rash, anaphylaxis, pruritus, chills, palpitations, flushing, dizziness, nausea, and dyspnea. Breathlessness, anaphylaxis and flushing are safety warning signs for Shenmai injection. Further prospective, multi-center, large-sample safety hospital-based monitoring of Shenmai injection was conducted in 32,358 cases. ADRs occurred in 30 patients with an incidence of 0.093% and were characterized by chest tightness, chills, itchy skin, palpitations, fever, nausea, dizziness, vomiting, flushing, paresthesia, anaphylactic reactions, cyanosis, rash and back pain. Patients with chronic pulmonary heart disease, thyroid disease, combined cerebrovascular disease, and continuous use of prostaglandin and cyclophosphopyridine before Shenmai injection combined with quinolones, penicillins, and TCM expectorants are prone to adverse reactions (Wang et al., 2015).

Xiang et al. (2017) reported the occurrence of the abovementioned adverse reactions. They noted that the adverse reactions caused by Shenmai injection mostly concentrated in the first 30 min of the medication process, and most of them were rapid-onset metamorphic reactions. Therefore, when Shenmai injection is used clinically, it is necessary to pay close attention to changes in patients’ signs in the first 30 min (Xiang et al., 2017).

Another study involving the use of Shenmai injection in 26 hospitals throughout China revealed that of 30,012 patients, 356 had adverse drug events (ADEs), and 45 had ADRs with an ADR incidence rate of 0.15%. Additionally, the adverse reactions disappeared after slowing the drip rate, discontinuing the drug, and treating the symptoms, which suggests that the safety of Shenmai injection is high (Wang et al., 2021).

With respect to the use of Shenmai injection, studies do not recommend the use of Shenmai injection with potassium chloride or vitamin C injection. The concomitant infusion of Shenmai injection with Xiangdan injection and LMWI should be avoided. Shenmai injection is contraindicated with pantoprazole sodium for injection (Deng and Xu, 2004; Zhang et al., 2008; Li and Xu, 2010).

In summary, compared with other therapeutic drugs, Shenmai injection has notable advantages in terms of safety, multi-target action, and efficacy in the treatment of VMC. In addition to its conventional antiviral, immune-regulating, and antioxidant properties, specifically in accelerating the clearance of oxygen free radicals, Shenmai injection improves microcirculation in VMC, enhances the myocardial contractility, and optimizes the myocardial metabolism. Therefore, Shenmai injection helps to prevent damage to myocardial cells in VMC and contributes to the repair of damaged myocardial cells. Furthermore, compared with traditional treatment methods for VMC, Shenmai injection combined with standard therapy has greater clinical efficacy. The findings of this study support the effectiveness of Shenmai injection in VMC (Shen, J. 2007; Liu, M. 2007; Shi et al., 2021).

### 4.1 Limitations

The limitations of this paper are as follows. First, too few randomized controlled trials were included in this study, and some of the articles were of low quality. More high-quality RCTs on this topic must be conducted to substantiate the efficacy of this treatment. Some of the randomized controlled trials in this article reported the use of blinding but did not specify whether blinding was used for patients, experimenters and evaluators or only for patients, which may have led to exaggerated results of the intervention group, biased results of the meta-analysis, and imperfect conclusion of this study. Although the studies in this paper mentioned the approximate medication for conventional treatment, the exact dosage and frequency of medication were not described in detail, which may have led to clinical heterogeneity. Clinical heterogeneity may increase due to different levels of care at different times and in different hospitals, but such limitations are difficult to avoid. The randomized controlled trials in this study are limited to China only, and the reference significance of this treatment regimen for populations in other countries or regions must be verified in larger clinical trials.

### 4.2 Prospects

This study highlights the inadequacy of the current clinical trials of Shenmai injection for the treatment of VMC. Future clinical studies should strengthen the application of blinding and ensure strict randomization in the trials. The type, dosage, and frequency of administration of conventional therapeutic drugs in the control group should also be clarified in clinical studies. Further in-depth studies on the target of action, clinical minimum starting concentration, and optimal treatment period of Shenmai injection to treat VMC are also necessary. Moreover, there are few reports on the side effects of Shenmai injection, which must be examined.

## 5 Conclusion

The combination of Shenmai injection with conventional therapy can improve the degree of myocardial damage, weaken the inflammatory response and improve the clinical efficacy of treatment for patients with VMC, which has a degree of safety. However, the quality of the current evidence is relatively low, and more high-quality studies are necessary to confirm the effectiveness of this treatment and provide more reliable evidence for its clinical application.

## Data Availability

The datasets presented in this study can be found in online repositories. The names of the repository/repositories and accession number(s) can be found in the article/Supplementary Material.

## Supplementary material

SUPPLEMENTARY FIGURE S1 Comparative forest plots of the TNF-α levels.

SUPPLEMENTARY FIGURE S2 Forest plots of the subgroup analysis at different doses (TNF-α level).

SUPPLEMENTARY FIGURE S3 Forest plots of the subgroup analysis at different durations of treatment (TNF-α level).

SUPPLEMENTARY FIGURE S4 Comparative forest plots of the TNF-α levels (removing sources of heterogeneity).

SUPPLEMENTARY FIGURE S5 Comparative forest plots of the IL-6 level.

SUPPLEMENTARY FIGURE S6 Forest plots of the subgroup analysis at different doses (IL-6 level).

SUPPLEMENTARY FIGURE S7 Comparative forest plots of the IL-6 level (removing sources of heterogeneity).

SUPPLEMENTARY FIGURE S8 Comparative forest plots of the adverse reactions.

SUPPLEMENTARY FIGURE S9 Funnel plot of the publication bias.

SUPPLEMENTARY FIGURE S10 Evidence quality analysis of the continuous variables.

SUPPLEMENTARY FIGURE S11 Evidence quality analysis of the bicategorical variables.
