# Supplementary material for: Efficacy and safety of Shenmai injection in the treatment of viral myocarditis: a systematic review and meta-analysis
Source: Front Pharmacol. 2024 Oct 25;15:1453946. doi: 10.3389/fphar.2024.1453946 (PMC11543498; doi:10.3389/fphar.2024.1453946)
Supplement: Supplementary file 1 [file Table1.docx]

**Supplementary Table S1 Search strategy**

| Databases | Search items | Numbers |
| --- | --- | --- |
| **PubMed** | #1:All Fields=“Myocarditis” OR “viral myocarditis” OR “Myocarditides” OR “Carditis”  #2: All Fields= “Shenmai Injection” OR “shenmai Injection” OR “ShenMai Injection”  #3.#1 and# 2 | 14 |
| **Web of Science** | #1: All Fields=“Myocarditis” OR “viral myocarditis” OR “Myocarditides” OR “Carditis”  #2: All Fields= “Shenmai Injection” OR “shenmai Injection” OR “ShenMai Injection”  #3: #1 and #2 | 10 |
| **Embase** | #1: Broad search=“Myocarditis” OR “viral myocarditis” OR “Myocarditides” OR “Carditis”  #2: Broad search= “Shenmai Injection” OR “shenmai Injection” OR “ShenMai Injection”  #3: #1 and #2 | 20 |
| **Cochrane Library** | #1:All Text=“Myocarditis” OR “viral myocarditis” OR “Myocarditides” OR “Carditis”  #2: All Text=“Shenmai Injection” OR “shenmai Injection” OR “ShenMai Injection”  #3: #1 and #2 | 33 |
| **CNKI** | #1:SU%='病毒性心肌炎'  #2:SU%='参麦注射液'  #3:#1 and #2 | 271 |
| **VIP** | U=（病毒性心肌炎）AND U=(参麦注射液) | 260 |
| **Wanfang Database** | #1:主题:(病毒性心肌炎)  #2:主题:(参麦注射液)  #3:#1 and #2 | 338 |
