# Supplementary figures and images for: Efficacy and safety of Shenmai injection in the treatment of viral myocarditis: a systematic review and meta-analysis
Source: Front Pharmacol. 2024 Oct 25;15:1453946. doi: 10.3389/fphar.2024.1453946 (PMC11543498; doi:10.3389/fphar.2024.1453946)

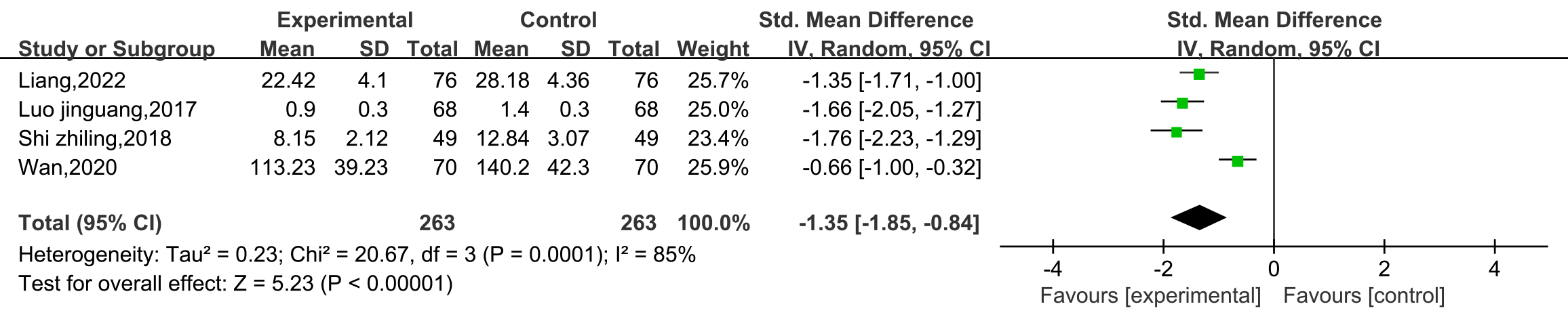

Supplement: Supplementary file 2 [file DataSheet1.zip › Supplementary Figures 1-11/Supplementary Figure 1-Comparative forest plots of TNF-a- level.tif]

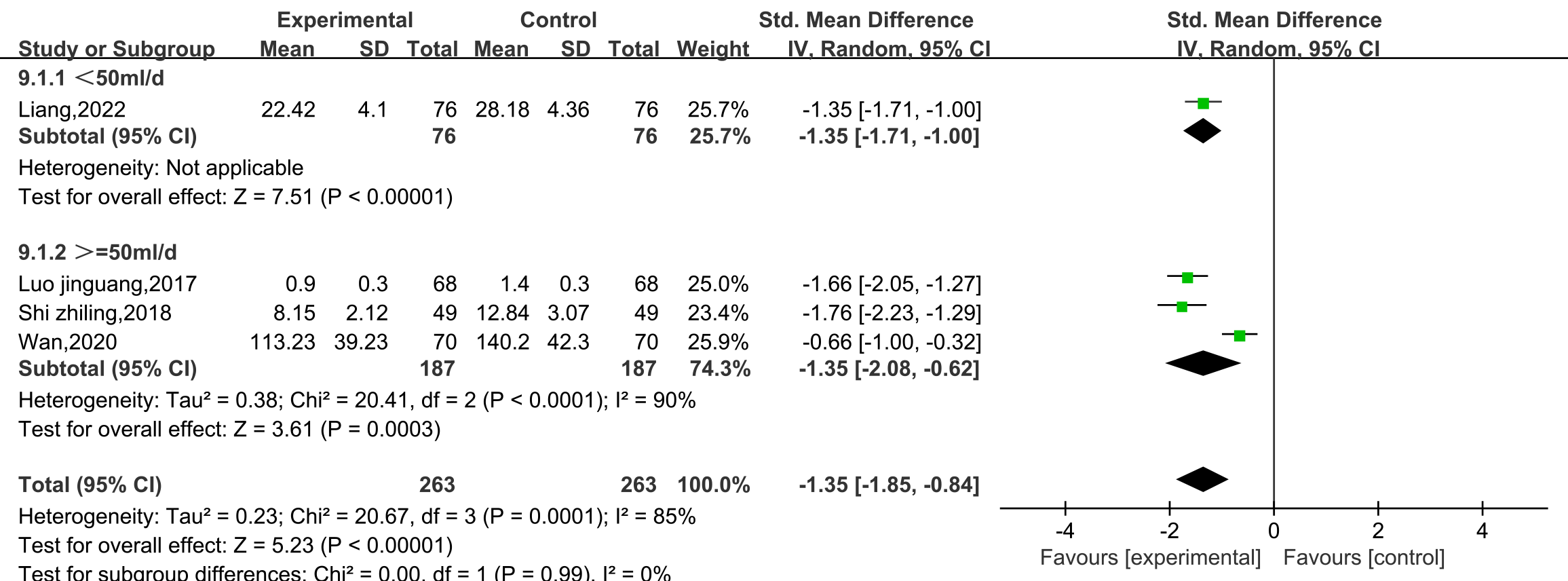

Supplement: Supplementary file 2 [file DataSheet1.zip › Supplementary Figures 1-11/Supplementary Figure 2-Forest plots of subgroup analysis at different doses ( TNF-a- level).tif]

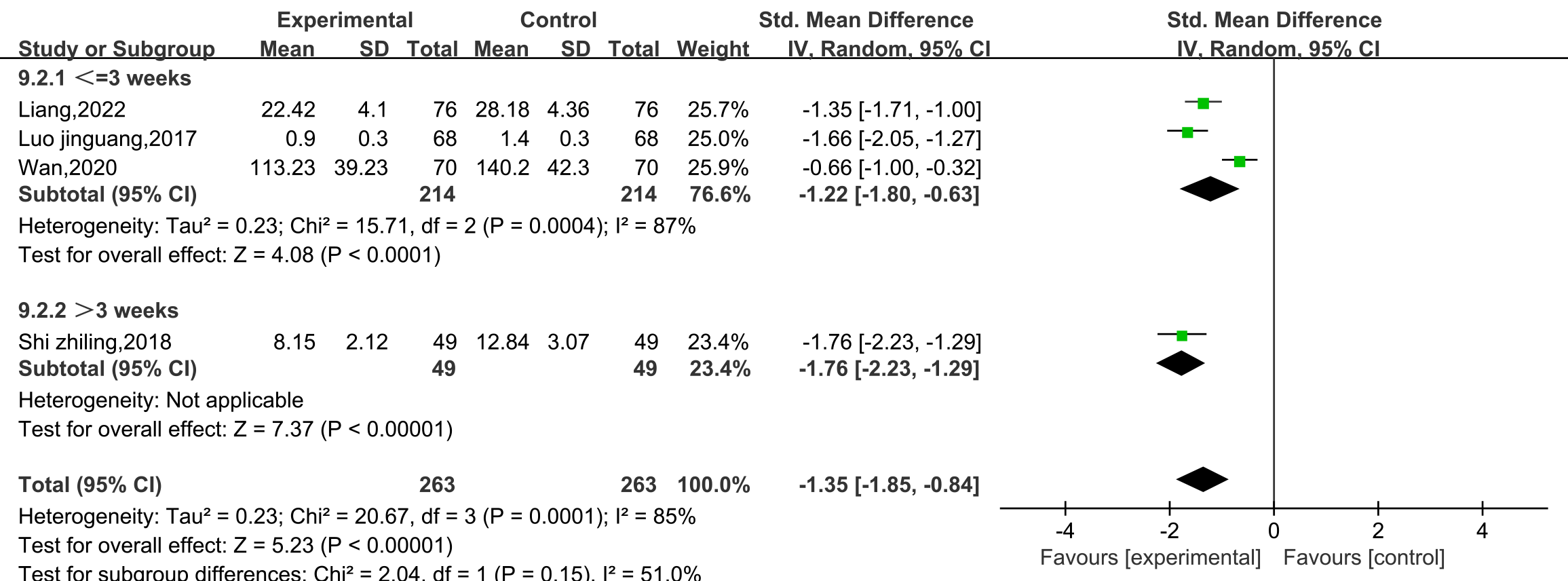

Supplement: Supplementary file 2 [file DataSheet1.zip › Supplementary Figures 1-11/Supplementary Figure 3-Forest plots of subgroup analysis at different duration of treatment (TNF-a- level).tif]

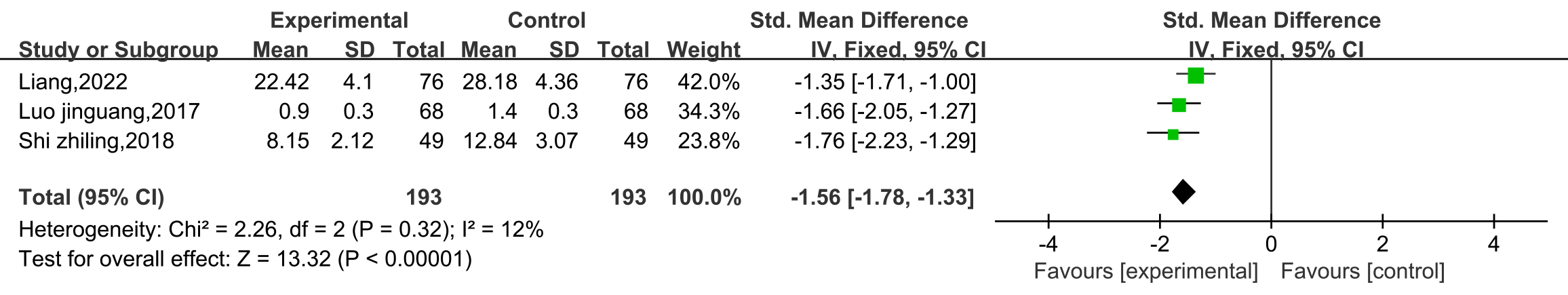

Supplement: Supplementary file 2 [file DataSheet1.zip › Supplementary Figures 1-11/Supplementary Figure 4-Comparative forest plots of TNF-a- leve(Remove sources of heterogeneity).tif]

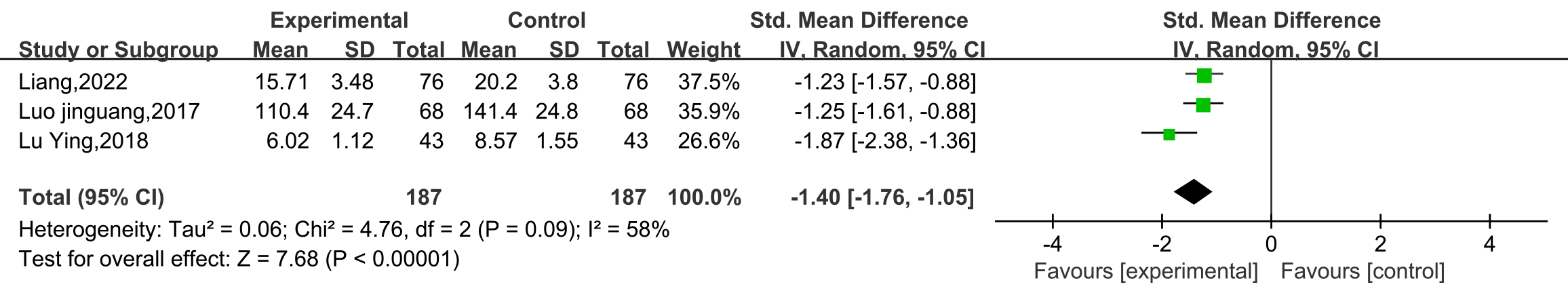

Supplement: Supplementary file 2 [file DataSheet1.zip › Supplementary Figures 1-11/Supplementary Figure 5-Comparative forest plots of IL-6 level.tif]

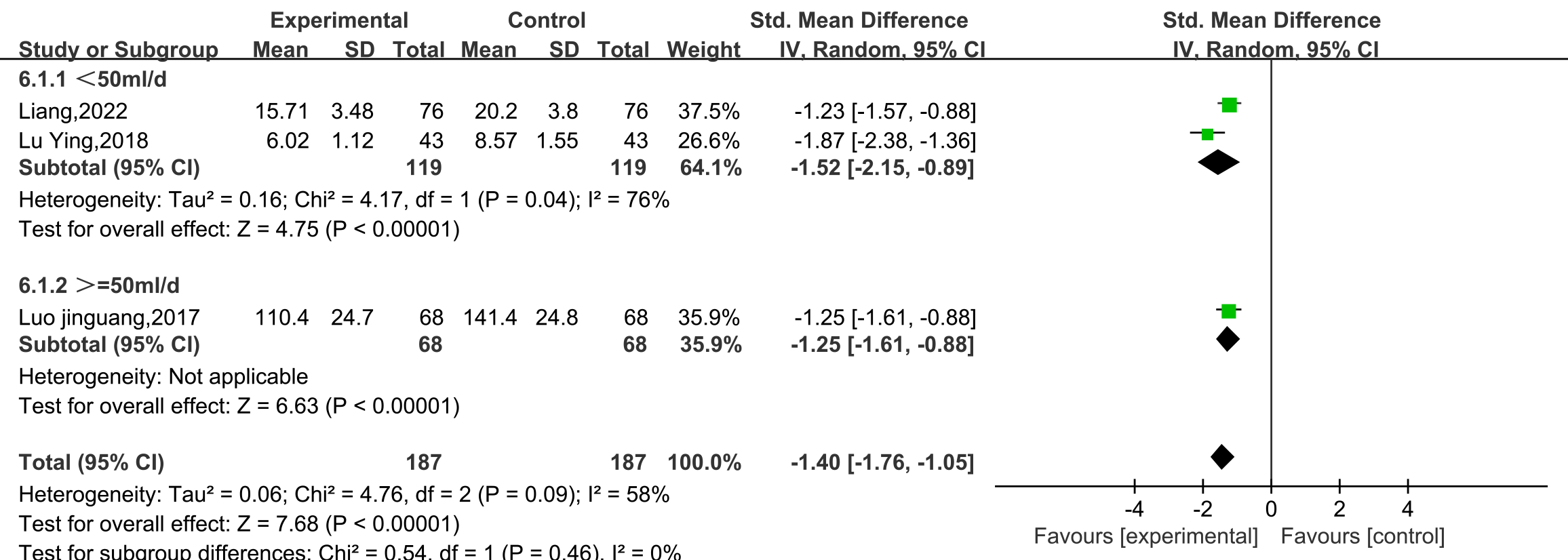

Supplement: Supplementary file 2 [file DataSheet1.zip › Supplementary Figures 1-11/Supplementary Figure 6-Forest plots of subgroup analysis at different doses (IL-6 level).tif]

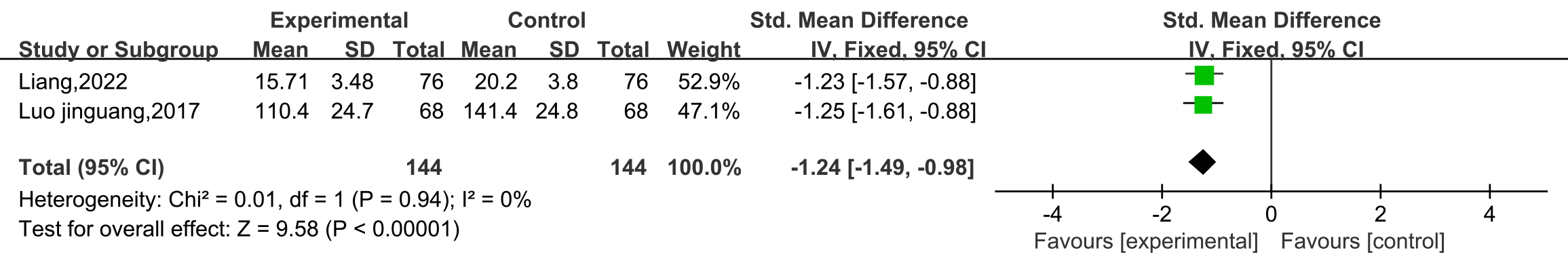

Supplement: Supplementary file 2 [file DataSheet1.zip › Supplementary Figures 1-11/Supplementary Figure 7-Comparative forest plots of IL-6 leve(Remove sources of heterogeneity).tif]

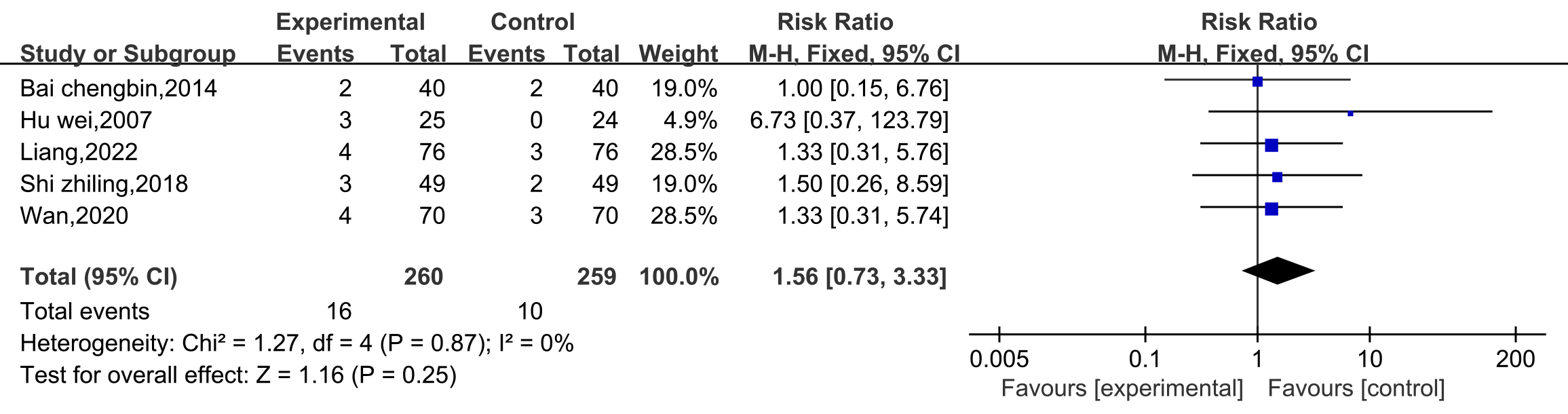

Supplement: Supplementary file 2 [file DataSheet1.zip › Supplementary Figures 1-11/Supplementary Figure 8-Comparative forest plots of adverse reactions.tif]

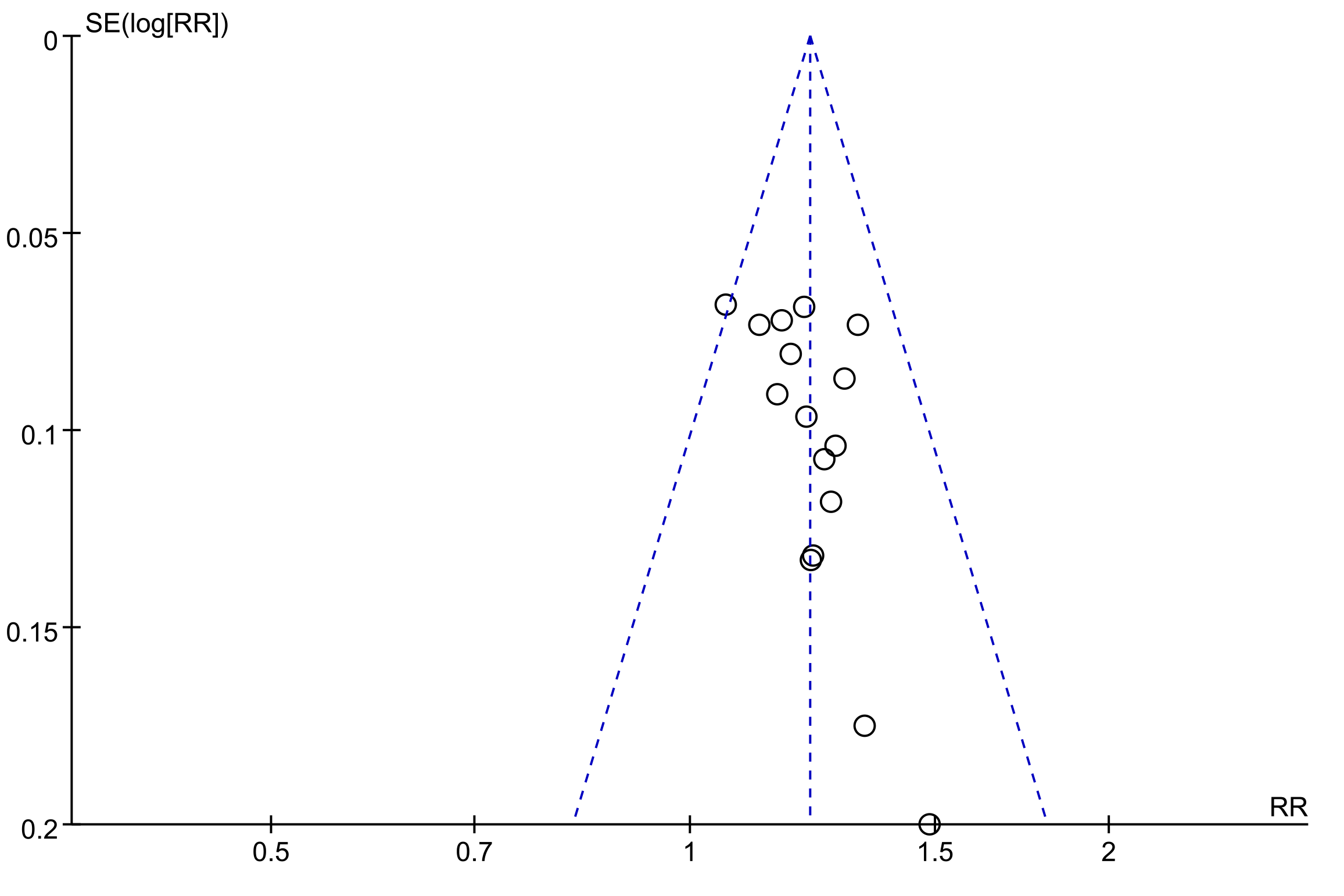

Supplement: Supplementary file 2 [file DataSheet1.zip › Supplementary Figures 1-11/Supplementary Figure 9-Funnel plot of Publication bias.tif]
